# Checkpoint TIPE2 Limits the Helper Functions of NK Cells in Supporting Antitumor CD8^+^ T Cells

**DOI:** 10.1002/advs.202207499

**Published:** 2023-02-19

**Authors:** Jiacheng Bi, Xiaomeng Jin, Chaoyue Zheng, Chen Huang, Chao Zhong, Xiaohu Zheng, Zhigang Tian, Haoyu Sun

**Affiliations:** ^1^ The CAS Key Laboratory of Quantitative Engineering Biology Shenzhen Institute of Synthetic Biology Shenzhen Institute of Advanced Technology Chinese Academy of Sciences Shenzhen 518055 P. R. China; ^2^ Institute of Systems Biomedicine School of Basic Medical Sciences Peking University Health Science Center Beijing 100191 P. R. China; ^3^ The CAS Key Laboratory of Innate Immunity and Chronic Disease School of Basic Medical Sciences, Division of Life Sciences and Medicine University of Science and Technology of China Hefei 230027 P. R. China; ^4^ Institute of Immunology University of Science and Technology of China Hefei 230027 P. R. China; ^5^ Research Unit of NK cell Study Chinese Academy of Medical Sciences Beijing 100864 P. R. China

**Keywords:** checkpoint inhibition, natural killer cells, Tbx21, Tnfaip8l2, tumor immunity

## Abstract

Natural killer (NK) cells not only are innate effector lymphocytes that directly participate in tumor surveillance but are also essential helpers in the antitumor CD8^+^ T‐cell response. However, the molecular mechanisms and potential checkpoints regulating NK cell helper functions remain elusive. Here, it is shown that the T‐bet/Eomes‐IFN‐*γ* axis in NK cells is essential for CD8^+^ T cell‐dependent tumor control, whereas T‐bet‐dependent NK cell effector functions are required for an optimal response to anti‐PD‐L1 immunotherapy. Importantly, NK cell‐expressed TIPE2 (tumor necrosis factor‐alpha‐induced protein‐8 like‐2) represents a checkpoint molecule for NK cell helper function, since *Tipe2* deletion in NK cells not only enhances NK‐intrinsic antitumor activity but also indirectly improves the antitumor CD8^+^ T cell response by promoting T‐bet/Eomes‐dependent NK cell effector functions. These studies thus reveal TIPE2 as a checkpoint for NK cell helper function, whose targeting might boost the antitumor T cell response in addition to T cell‐based immunotherapy.

## Graphical abstract

Natural killer (NK) cells are essential helpers for antitumor CD8^+^ T‐cell response. NK cell‐expressed TIPE2 (tumor necrosis factor‐alpha‐induced protein‐8 like‐2) represents a checkpoint molecule for NK cell helper function, since *Tipe2* deletion in NK cells not only enhances NK‐intrinsic antitumor activity, but also indirectly improves antitumor CD8^+^ T cell response by promoting T‐bet/Eomes‐dependent NK cell effector functions.

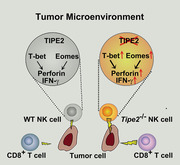

## 1 Introduction

Natural killer (NK) cells are cytotoxic innate lymphocytes that play essential roles in first‐line tumor surveillance.^[^ ^1^ ^]^ Both IFN‐*γ* production and cytotoxicity represent the major antitumor effector functions of NK cells, which are governed by the T‐box transcription factors T‐bet and Eomes.^[^ ^2^, ^3^ ^]^ In addition to direct tumor surveillance, NK cells also display helper function to orchestrate the subsequent CD8^+^ T cell antitumor response.^[^ ^4^, ^5^ ^]^ At the first step of antitumor CD8^+^ T cell response, tumor antigen released from dead tumor cells killed by NK cells could be acquired by dendritic cells (DCs) for presentation to CD8^+^ T cells.^[^ ^6^ ^]^ Next, NK cells facilitate DC maturation by preferential cytotoxicity against immature DCs,^[^ ^7^ ^]^ as well as by secretion of IFN‐*γ* and TNF‐*α* for upregulation of costimulatory molecules and production of IL‐12 by DCs.^[^ ^8^ ^]^ In addition, tumor recognition by CD8^+^ T cells also required NK cells’ help, since NK cell‐derived IFN‐*γ* upregulates MHC I expression on tumor cells.^[^ ^9^ ^]^ On the other hand, NK cells play an important role in CD8^+^ T cell‐dependent PD‐1/PD‐L1 checkpoint immunotherapy. Not only the response to anti‐PD‐1 therapy correlates with the levels of tumor‐associated NK cells,^[^ ^10^ ^]^ depletion of NK cells also compromises the improved survival by anti‐PD‐L1 therapy in tumor‐bearing mice,^[^ ^11^ ^]^ and activated human NK cells could sensitize tumor cells to PD‐1 blockade therapy in vitro.^[^ ^12^ ^]^ Despite the above‐mentioned accumulating evidence of NK cell helper functions in supporting the CD8^+^ T cell antitumor response, the underlying molecular mechanisms remain poorly defined. On the other hand, studies on NK cell helper functions suggest that stimulation of NK cell activity might indirectly promote the antitumor CD8^+^ T cell response. However, the molecular checkpoints governing NK cell helper function remain elusive.

Checkpoint molecules, including inhibitory cell surface receptors and intracellular regulatory molecules, mediate tumor‐associated NK cell dysfunction,^[^ ^11^, ^13^ ^]^ a status characterized by diminished effector functions, as well as by downregulation of the key transcription factors T‐bet and Eomes.^[^ ^14^ ^]^ Therefore, checkpoint molecules represent potential targets for improving NK cell antitumor immunity. TIPE2 (tumor necrosis factor‐alpha‐induced protein‐8 like‐2) represents an intracellular checkpoint molecule for both innate and adaptive immune responses.^[^ ^15^ ^]^ *Tipe2*‐deficient immune cells are hyper‐sensitive to Toll‐like receptor (TLR) and T cell receptor (TCR) stimulation.^[^ ^15^ ^]^ During infection, *Tipe2* knockout cells show enhanced phagocytosis and oxidative burst.^[^ ^16^ ^]^ In lipopolysaccharide‐stimulated macrophages, *Tipe2* deficiency increases expression of iNOS expression and NO production, and decreases expression of arginase I and urea production.^[^ ^17^ ^]^ In mouse tumor models, global loss of TIPE2 suppresses tumor growth.^[^ ^18^ ^]^ Loss of TIPE2 promotes the expression of antitumoral mediators and reduces the expression of protumoral mediators in myeloid‐derived suppressor cells.^[^ ^18^ ^]^ In addition to these studies, a recent study indicates that TIPE2 is also a checkpoint limiting NK cell activity, since NK‐specific *Tipe2* knockout mice are more resistant to tumor challenge.^[^ ^19^ ^]^ Deletion of *Tipe2* promotes NK cell functional maturation and antitumor immunity by increasing IL‐15‐mTOR signaling activity.^[^ ^19^ ^]^ In this study, we revealed that NK cells were required for an optimal antitumor CD8^+^ T cell immune response via the T‐bet/Eomes‐IFN‐*γ* axis and promoted anti‐PD‐L1 immunotherapy through T‐bet‐dependent NK cell antitumor effector functions. Furthermore, NK‐specific *Tipe2* deletion not only prevented the exhaustion of tumor‐infiltrating NK cells but also indirectly improved the antitumor CD8^+^ T cell response by promoting T‐bet/Eomes‐dependent NK cell effector functions. Therefore, the checkpoint molecule TIPE2 not only suppresses NK‐intrinsic antitumor effector functions but also limits NK cell helper function in supporting the antitumor CD8^+^ T cell response.

## 2 Results

### 2.1 NK Cells are Required for an Optimal Antitumor CD8^+^ T Cell Immune Response

**Figure 1:**
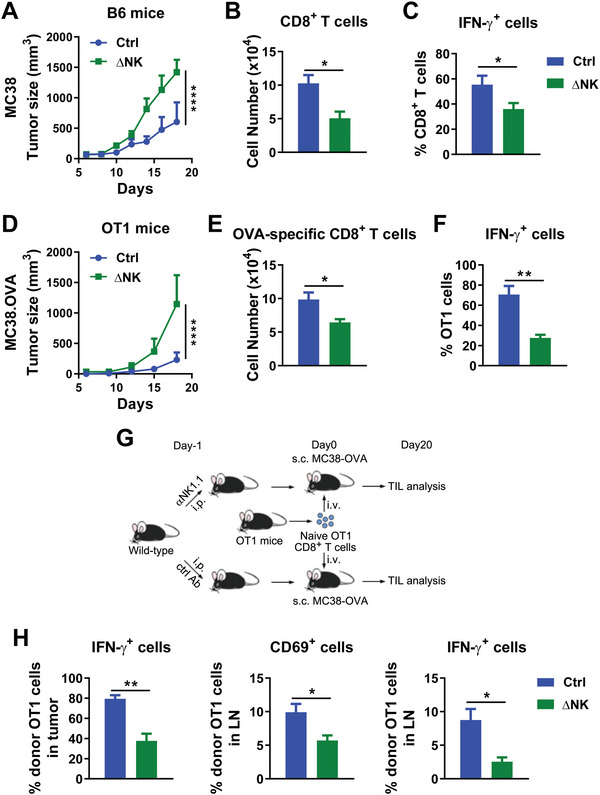
Depletion of NK cells leads to functional impairment of antitumor CD8^+^ T cells. A) MC38 tumor growth in NK‐depleted or control WT B6 mice. B) CD8^+^ T cell numbers infiltrating per gram tumor mass, C) as well as percentages of IFN‐*γ*‐producing cells among tumor‐infiltrating CD8^+^ T cells upon PMA and Ionomycin stimulation ex vivo on day 22 post‐tumor challenge from (A). D) MC38‐OVA tumor growth in NK‐depleted or control OT1 mice. E) OT1 CD8^+^ T cell numbers infiltrating per gram tumor mass, F) as well as percentages of IFN‐*γ*‐producing cells among tumor‐infiltrating CD8^+^ T cells upon PMA and Ionomycin stimulation ex vivo on day 22 post‐tumor challenge from (D). G) Scheme of splenic CD62L^+^CD44^−^ naïve OT1 cell transfer into NK‐depleted or control WT B6 mice challenged with MC38‐OVA tumor cells. H) Percentages of IFN‐*γ*‐producing cells among OT1 cells in the tumor tissue upon PMA and Ionomycin stimulation ex vivo or CD69 expression and percentages of IFN‐*γ*‐producing cells of OT1 cells in the TdLN upon PMA and Ionomycin stimulation ex vivo from (G). A–C) Data are representative of at least three independent experiments (*n* = 6). D–H) Data are representative of two independent experiments (*n* = 6). B,C,E,F,H) Data are mean values ± SEM. *p < 0.05, **p < 0.005 (two‐tailed Student's *t*‐test). A,D) ****p < 0.0001 (two‐way ANOVA).

To investigate NK cell helper function in supporting CD8^+^ T cell‐dependent tumor control, we set out to determine the effects of NK cell depletion on tumor growth and the CD8^+^ T cell antitumor response. The absence of NK cells compromised host control of tumor growth, since depletion of NK cells led to faster MC38 tumor growth in vivo (**Figure** **1**A). The absence of NK cells also decreased the number of tumor‐infiltrating CD8^+^ T cells (Figure 1B) and their IFN‐*γ* production (Figure 1C), indicating that NK cells are required for an optimal antitumor CD8^+^ T cell response. We next investigated the role of NK cells in the tumor antigen‐specific CD8^+^ T cell response. Depletion of NK cells resulted in accelerated MC38‐OVA tumor growth in OT1 mice (Figure 1D), as well as reduced tumor‐infiltrating OT1 CD8^+^ T cells (Figure 1E) and compromised production of IFN‐*γ* by OT1 CD8^+^ T cells (Figure 1F). Furthermore, we adoptively transferred naïve OT1 CD8^+^ T cells into NK‐depleted or control WT mice following MC38‐OVA tumor challenge to determine the role of NK cells in immune competent hosts (Figure 1G). Donor‐derived OT1 CD8^+^ T cells infiltrating the tumor produced less IFN‐*γ* in NK‐depleted hosts than in control hosts (Figure 1H). Donor‐derived OT1 CD8^+^ T cells in TdLNs (tumor‐draining lymph nodes) also displayed decreased activation, as evidenced by the reduced expression of CD69 and IFN‐*γ* in NK‐depleted hosts compared with control hosts (Figure 1H). These data together demonstrated that NK cells are required for an optimal tumor antigen‐specific CD8^+^ T cell response.

### 2.2 Deletion of *Tipe2* in NK Cells Improves the Antitumor Function of CD8^+^ T Cells

**Figure 2:**
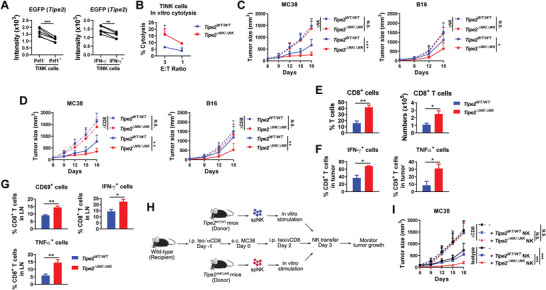
Deletion of *Tipe2* in NK cells improves the antitumor function of CD8^+^ T cells. A) Tumor‐infiltrating NK cells were analyzed for EGFP intensity among perforin‐ or IFN‐*γ*‐positive or ‐negative cells in *Tipe2* reporter mice 20 days post‐challenge with MC38 tumor cells. **p < 0.005, ***p < 0.001 (paired Student's *t*‐test). B) Tumor‐infiltrating NK cells were purified for an in vitro cytolytic assay against YAC‐1 cells at the indicated effector:target ratio. *p < 0.05 (one‐way ANOVA). C) MC38 or B16 tumor growth in NK‐depleted or control *Tipe2* ^WT/ WT^ mice or *Tipe2* ^ΔNK/ΔNK^ mice. D) MC38 or B16 tumor growth in CD8^+^ cell‐depleted or control *Tipe2* ^WT/ WT^ mice or *Tipe2* ^ΔNK/ΔNK^ mice. C,D) *n* = 6. *p < 0.05, **p < 0.005, ***p < 0.001 (two‐way ANOVA). E) Percentages of CD8^+^ cells among CD3^+^NK1.1^−^ T cells, as well as CD8^+^ T cell numbers of tumor‐infiltrating lymphocytes per gram tumor tissue 19 days post MC38 tumor challenge. F) Percentages of IFN‐*γ*‐ and TNF‐*α*‐producing cells among CD8^+^ T cells in the tumor tissue upon PMA and Ionomycin stimulation ex vivo 19 days post MC38 tumor challenge. G) CD69, IFN‐*γ*, and TNF‐*α* expression in CD8^+^ T cells in the TdLN 17 days post MC38 tumor challenge. IFN‐*γ* and TNF‐*α* were analyzed after PMA and Ionomycin stimulation ex vivo. E–G) *p < 0.05, **p < 0.005 (two‐tailed Student's *t*‐test). H) Scheme of splenic NK cell transfer into CD8^+^ cell‐depleted or control WT B6 mice challenged with MC38 tumor cells. I) Tumor growth in (H). “‐” in the legend indicates no transfer. ***p < 0.001 (two‐way ANOVA). A–I) Data are mean values ± SEM representative of two independent experiments.

Based on the helper role of NK cells in supporting the antitumor CD8^+^ T cell response we observed above, we further investigated the potential regulator of NK cell helper function. We previously reported TIPE2 as a checkpoint molecule in NK cells limiting their functional maturation and antitumor immunity.^[^ ^19^ ^]^ We wondered whether TIPE2 in NK cells might also contribute to the regulation of NK cell help for the antitumor CD8^+^ T cell response. Since perforin‐dependent cytolytic activity represents the major antitumor effector function of NK cells,^[^ ^1^ ^]^ we first analyzed the correlation of *Tipe2* (EGFP) versus perforin expression in tumor‐infiltrating NK cells in MC38 tumor‐bearing *Tipe2* reporter mice. We found that perforin‐negative NK cells expressed higher levels of EGFP than perforin‐positive NK cells (**Figure** **2**A). Similarly, IFN‐*γ*‐negative NK cells expressed higher levels of EGFP (Figure 2A). Consistently, tumor‐infiltrating NK cells displayed higher cytolytic activity in *Tipe2^ΔNK/ΔNK^* mice (Figure 2B) in which *Tipe2* gene expression was deficient in NK cells with no reduction in the NK cell population size.^[^ ^19^ ^]^ These data confirmed our previous findings that TIPE2 suppresses the antitumor functions of tumor‐infiltrating NK cells.^[^ ^19^ ^]^ In line with this, depletion of NK cells by anti‐NK1.1 monoclonal antibodies abrogated the tumor‐suppressing effects of NK‐specific TIPE2 deficiency in *Tipe2^ΔNK/ΔNK^* mice compared with control mice both in the MC38 model and in the B16 model (Figure 2C) indicating that NK cells were essential in the improved antitumor response in *Tipe2^ΔNK/ΔNK^* mice. More importantly, depletion of CD8^+^ T cells by anti‐CD8 monoclonal antibodies also compromised the tumor‐suppressing effects of NK‐specific TIPE2 deficiency in *Tipe2^ΔNK/ΔNK^* mice (Figure 2D), indicating that CD8^+^ T cells were also required for the improved antitumor response in *Tipe2^ΔNK/ΔNK^* mice, suggesting that *Tipe2* deletion in NK cells might indirectly promote the antitumor CD8^+^ T cell response. Indeed, analysis of tumor‐infiltrating lymphocytes showed a higher percentage of CD8^+^ T cells among total CD3^+^NK1.1^−^ T cells, as well as higher absolute numbers of CD8^+^ T cells in the tumor tissue from *Tipe2^ΔNK/ΔNK^* mice over control mice (Figure 2E). Tumor‐infiltrating CD8^+^ T cells from *Tipe2^ΔNK/ΔNK^* mice also displayed increased production of IFN‐*γ* and TNF‐*α* compared with those from control mice (Figure 2F). In addition, CD8^+^ T cells in the TdLNs from *Tipe2^ΔNK/ΔNK^* mice expressed higher levels of CD69, IFN‐*γ*, and TNF‐*α* than those from control mice (Figure 2G). To confirm the role of *Tipe2^−/−^* NK cells in supporting the antitumor function of CD8^+^ T cells, we adoptively transferred NK cells into MC38 tumor‐bearing mice to determine the role of NK‐expressed TIPE2 in tumor surveillance and its relationship with CD8^+^ T cells (Figure 2H). Transferring *Tipe2* ^−/−^ NK cells into MC38 tumor‐bearing mice conferred superior tumor control over wild‐type NK cells, confirming the improved tumor control by *Tipe2^−/−^* NK cells (Figure 2I). Importantly, depletion of CD8^+^ T cells abrogated such effects, as transferring *Tipe2* ^−/−^ NK cells displayed comparable effects on tumor growth as transferring wild‐type NK cells in recipient mice treated with anti‐CD8 antibody (Figure 2I). These results demonstrated that deletion of *Tipe2* in NK cells not only promotes NK cell antitumor activity but also indirectly improves the antitumor function of CD8^+^ T cells.

### 2.3 Deletion of *Tipe2* in NK Cells Improves the Tumor Antigen‐Specific CD8^+^ T Cell Response

**Figure 3:**
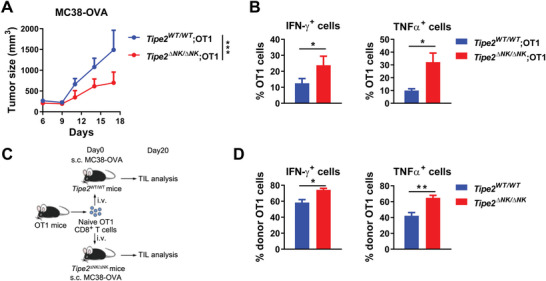
Deletion of *Tipe2* in NK cells improves the tumor antigen‐specific CD8^+^ T cell response. A) MC38‐OVA tumor growth in *Tipe2* ^ΔNK/ΔNK^; OT1 mice or control OT1 mice. B) Percentages of IFN‐*γ*‐ or TNF‐*α*‐producing cells among tumor‐infiltrating OT1 cells upon PMA and Ionomycin stimulation ex vivo 23 days post‐tumor challenge from (A). C) Scheme of splenic CD62L^+^CD44^−^ naïve OT1 cell transfer into *Tipe2* ^ΔNK/ΔNK^ or *Tipe2* ^WT/WT^ mice challenged with MC38‐OVA tumor cells. D) Percentages of IFN‐*γ*‐ or TNF‐*α*‐producing cells among tumor‐infiltrating donor‐derived OT1 cells upon PMA and Ionomycin stimulation ex vivo 23 days post‐tumor challenge from (C). A,B,D) Data are mean values ± SEM representative of two independent experiments (*n* = 6). *p < 0.05, **p < 0.005, ***p < 0.001 (two‐way ANOVA for (A), two‐tailed Student's *t*‐test for (B) and (D)).

The above data prompted us to further investigate the role of NK‐expressed TIPE2 in the tumor antigen‐specific CD8^+^ T cell response. We observed reduced growth of MC38‐OVA tumors in *Tipe2^ΔNK/ΔNK^* OT1 mice compared with control OT1 mice (**Figure** **3**A). The production of IFN‐*γ* and TNF‐*α* by tumor‐infiltrating OT1 CD8^+^ T cells was higher in *Tipe2^ΔNK/ΔNK^* OT1 mice than in control OT1 mice (Figure 3B). Adoptive transfer of CD62L^+^CD44^−^ naïve OT1 CD8^+^ T cells to *Tipe2^ΔNK/ΔNK^* mice or control mice bearing MC38‐OVA tumors showed that donor‐derived tumor‐infiltrating OT1 CD8^+^ T cells in *Tipe2^ΔNK/ΔNK^* mice expressed higher levels of IFN‐*γ* and TNF‐*α* (Figure 3C,D). These data indicated that deletion of *Tipe2* in NK cells improves the tumor antigen‐specific CD8^+^ T cell response.

### 2.4 TIPE2 Deficiency Increases T‐bet and Eomes Activity in NK Cells

**Figure 4:**
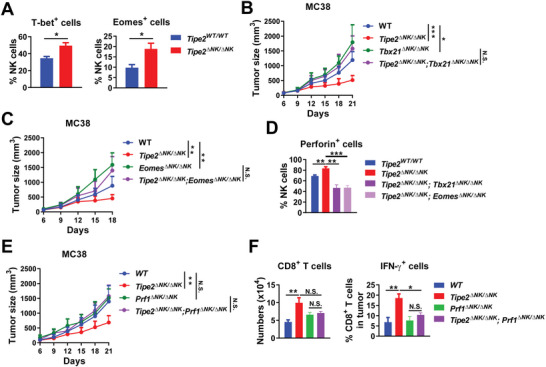
TIPE2 deficiency increases T‐bet and Eomes activity in NK cells. A) Percentages of T‐bet‐ or Eomes‐expressing cells among tumor‐infiltrating NK cells from MC38 tumor‐bearing *Tipe2* ^WT/ WT^ mice or *Tipe2* ^ΔNK/ΔNK^ mice 14 days post‐tumor challenge. B) MC38 tumor growth in WT, *Tipe2* ^ΔNK/ΔNK^, *Tbx21* ^ΔNK/ΔNK^, or *Tipe2* ^ΔNK/ΔNK^; *Tbx21* ^ΔNK/ΔNK^ mice. C) MC38 tumor growth in WT, *Tipe2* ^ΔNK/ΔNK^, *Eomes* ^ΔNK/ΔNK^, or *Tipe2* ^ΔNK/ΔNK^; *Eomes* ^ΔNK/ΔNK^ mice. D) Percentages of perforin‐expressing cells among tumor‐infiltrating NK cells from MC38‐bearing *Tipe2* ^WT/ WT^, *Tipe2* ^ΔNK/ΔNK^, *Tipe2* ^ΔNK/ΔNK^; *Tbx21* ^ΔNK/ΔNK^, or *Tipe2* ^ΔNK/ΔNK^; *Eomes* ^ΔNK/ΔNK^ mice 29 days post‐tumor challenge. E) MC38 tumor growth in WT, *Tipe2* ^ΔNK/ΔNK^, *Prf1* ^ΔNK/ΔNK^, or *Tipe2* ^ΔNK/ΔNK^; *Prf1* ^ΔNK/ΔNK^ mice. F) Numbers of tumor‐infiltrating CD8^+^ T cells per gram of tumor tissue and percentages of IFN‐*γ*‐producing cells among tumor‐infiltrating CD8^+^ T cells upon PMA and Ionomycin stimulation ex vivo in MC38 tumor‐bearing WT, *Tipe2* ^ΔNK/ΔNK^, *Prf1* ^ΔNK/ΔNK^, or *Tipe2* ^ΔNK/ΔNK^; *Prf1* ^ΔNK/ΔNK^ mice 28 days post‐tumor challenge. A–F) Data are mean values ± SEM representative of two independent experiments (*n* = 6). *p < 0.05, **p < 0.005, ***p < 0.001 (two‐tailed Student's *t*‐test for (A, D, F); two‐way ANOVA for (B, C, E)).

T‐bet and Eomes are key transcription factors for NK cell effector functions and antitumor immunity.^[^ ^2^, ^3^ ^]^ To investigate the underlying mechanisms of action by *Tipe2^−/‐^* NK cells in supporting the CD8^+^ T cell antitumor response, we analyzed the expression of T‐bet and Eomes in tumor‐infiltrating NK cells. NK cells expressed higher levels of both T‐bet and Eomes in the absence of TIPE2 than in the presence of TIPE2 (**Figure** **4**A), suggesting that TIPE2 expression might suppress T‐bet and Eomes expression in tumor‐infiltrating NK cells. NK‐specific T‐bet or Eomes deficiency led to accelerated tumor growth in mice (Figure 4B,C) indicative of the essential roles of NK‐expressed T‐bet and Eomes in tumor control. Furthermore, although *Tipe2^ΔNK/ΔNK^* mice displayed superior tumor control over wild‐type mice, MC38 tumor growth was similar between *Tbx21^ΔNK/ΔNK^* mice and *Tipe2^ΔNK/ΔNK^; Tbx21^ΔNK/ΔNK^* mice (Figure 4B) and was also similar between *Eomes^ΔNK/ΔNK^* mice and *Tipe2^ΔNK/ΔNK^; Eomes^ΔNK/ΔNK^* mice (Figure 4C), indicating that the benefits on tumor control from NK‐specific *Tipe2* deletion were dependent on NK‐expressed T‐bet and Eomes. Therefore, TIPE2 deficiency in NK cells might improve antitumor immunity by increasing the expression of T‐bet and Eomes.

Next, we wondered how the increased expression of T‐bet and Eomes in *Tipe2^−/−^* NK cells promotes CD8^+^ T cell‐dependent antitumor immunity. T‐bet and Eomes are transcription factors essential for the expression of effector molecules in NK cells. We already showed that *Tipe2^−/−^* tumor‐infiltrating NK cells displayed enhanced cytolytic activity (Figure 2B). Since perforin‐dependent cytolytic activity represents the major antitumor effector function of NK cells,^[^ ^1^ ^]^ we determined the expression of perforin in tumor‐infiltrating NK cells. While tumor‐infiltrating NK cells expressed higher levels of perforin in *Tipe2^ΔNK/ΔNK^* mice than in wild‐type mice, the elevated perforin expression by *Tipe2* deletion was compromised upon further deletion of *Tbx21* or *Eomes*, as in *Tipe2^ΔNK/ΔNK^; Tbx21^ΔNK/ΔNK^* mice and in *Tipe2^ΔNK/ΔNK^; Eomes^ΔNK/ΔNK^* mice (Figure 4D), confirming that both transcription factors are required for the improved NK cell antitumor effector functions in *Tipe2^ΔNK/ΔNK^* mice. In the presence of TIPE2, tumor growth was not affected significantly by conditional knockout of perforin in NK cells (*Prf1^ΔNK/ΔNK^* mice) (Figure 4E), possibly due to the minimal contribution to overall tumor control by perforin‐dependent antitumor effector functions of the exhausted tumor‐infiltrating NK cells, which was diminished by TIPE2. In addition, while the improved tumor control in *Tipe2^ΔNK/ΔNK^* mice was compromised by further deletion of *Prf1* in *Tipe2^ΔNK/ΔNK^; Prf1^ΔNK/ΔNK^* mice, tumor growth in *Tipe2^ΔNK/ΔNK^; Prf1^ΔNK/ΔNK^* mice was similar to tumor growth in *Prf1^ΔNK/ΔNK^* mice (Figure 4E), suggesting that the improved tumor control by NK‐specific *Tipe2* deletion requires perforin‐dependent NK cell antitumor effector functions. More importantly, the improved production of IFN‐*γ* by tumor‐infiltrating CD8^+^ T cells in *Tipe2^ΔNK/ΔNK^* mice was compromised by further deletion of *Prf1* in *Tipe2^ΔNK/ΔNK^; Prf1^ΔNK/ΔNK^* mice, while both cell numbers and IFN‐*γ* production of tumor‐infiltrating CD8^+^ T cells were comparable between *Tipe2^ΔNK/ΔNK^; Prf1^ΔNK/ΔNK^* mice and *Prf1^ΔNK/ΔNK^* mice (Figure 4F), indicating that the improved help of *Tipe2^−/−^* NK cells for the antitumor function of CD8^+^ T cells was dependent on NK cell antitumor effector functions.

Taken together, these results demonstrated that the improved tumor control in *Tipe2^ΔNK/ΔNK^* mice was mediated by both T‐bet and Eomes‐dependent NK cell effector functions, suggesting that TIPE2 deficiency in NK cells might unleash CD8^+^ T cell‐dependent antitumor immunity by increasing the effector functions of NK cells through promoting the activity of T‐bet and Eomes.

### 2.5 T‐bet, Eomes, or IFN‐*γ* in NK Cells is Required for an Optimal Antitumor CD8^+^ T Cell Immune Response

**Figure 5:**
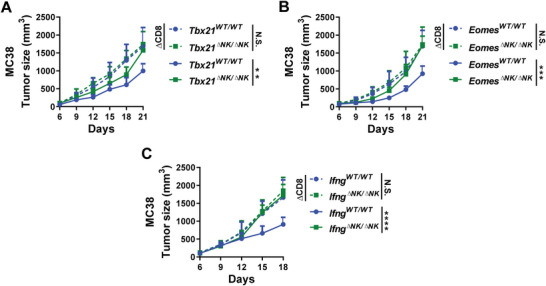
Deficiency of T‐bet, Eomes, or IFN‐*γ* in NK cells attenuates the antitumor function of CD8^+^ T cells. A) MC38 tumor growth in CD8^+^ cell‐depleted or control *Tbx21* ^WT/ WT^ mice or *Tbx21* ^ΔNK/ΔNK^ mice. B) MC38 tumor growth in CD8^+^ cell‐depleted or control *Eomes* ^WT/ WT^ mice or *Eomes* ^ΔNK/ΔNK^ mice. C) MC38 tumor growth in CD8^+^ cell‐depleted or control *Ifng* ^WT/ WT^ mice or *Ifng* ^ΔNK/ΔNK^ mice. A–C) Data are mean values ± SEM representative of two independent experiments (*n* = 6). **p < 0.005, ***p < 0.001, ****p < 0.0001 (two‐way ANOVA).

To investigate the mechanisms of the role of NK cells in supporting the CD8^+^ T cell‐dependent antitumor response, we set out to determine the relationship between NK cell‐expressed T‐bet and Eomes‐dependent tumor control and CD8^+^ T cell‐dependent antitumor immunity. Although we already observed accelerated tumor growth in both *Tbx21^ΔNK/ΔNK^* mice and *Eomes^ΔNK/ΔNK^* mice compared with control mice, we found that both *Tbx21^ΔNK/ΔNK^* mice and *Eomes^ΔNK/ΔNK^* mice displayed similar tumor growth as control mice after CD8^+^ T cell depletion (**Figure** **5**A,B), indicating that NK‐expressed T‐bet and Eomes‐mediated tumor control was dependent on CD8^+^ T cells.

As the major effector cytokine in T‐bet and Eomes‐dependent NK cell effector functions,^[^ ^2^, ^3^ ^]^ IFN‐*γ* plays a role in facilitating DC maturation and antigen presentation for CD8^+^ T cell activation.^[^ ^20^, ^21^, ^22^ ^]^ We hypothesized that IFN‐*γ* might play a role in NK cell help for the antitumor CD8^+^ T cell response. MC38 tumor growth was significantly accelerated in *Ifng^ΔNK/ΔNK^* mice, closely resembling tumor growth in CD8^+^ T cell‐depleted control mice (Figure 5C). In addition, MC38 tumor growth was comparable between CD8^+^ T cell‐depleted *Ifng^ΔNK/ΔNK^* mice and CD8^+^ T cell‐depleted control mice (Figure 5C), indicating that the beneficial effects on tumor control by IFN‐*γ* production of NK cells were dependent on CD8^+^ T cells. These data demonstrated that NK‐expressed IFN‐*γ* is required for the support of NK cells in CD8^+^ T cell‐dependent tumor control.

### 2.6 *Tipe2* Deletion in NK Cells Improves the Therapeutic Efficacy of PD‐L1 Blockade

**Figure 6:**
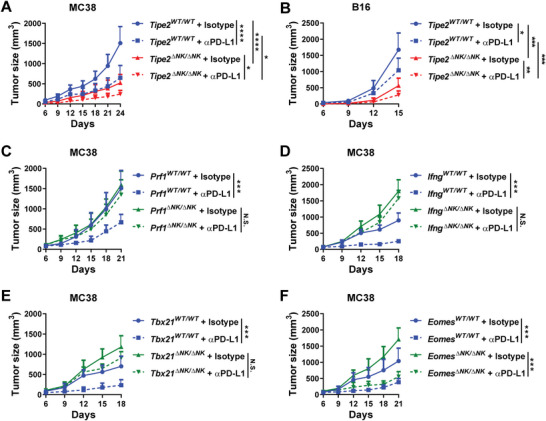
Deletion of *Tipe2* in NK cells improves the therapeutic efficacy of PD‐L1 blockade. A) MC38 or B) B16 tumor growth in *Tipe2* ^WT/ WT^ mice or *Tipe2* ^ΔNK/ΔNK^ mice treated with anti‐PD‐L1 antibody or isotype control antibody. C) MC38 tumor growth in *Prf1* ^WT/ WT^ mice or *Prf1* ^ΔNK/ΔNK^ mice treated with anti‐PD‐L1 antibody or isotype control antibody. D) MC38 tumor growth in *Ifng* ^WT/ WT^ mice or *Ifng* ^ΔNK/ΔNK^ mice treated with anti‐PD‐L1 antibody or isotype control antibody. E) MC38 tumor growth in *Tbx21* ^WT/ WT^ mice or *Tbx21* ^ΔNK/ΔNK^ mice treated with anti‐PD‐L1 antibody or isotype control antibody. F) MC38 tumor growth in *Eomes* ^WT/ WT^ mice or *Eomes* ^ΔNK/ΔNK^ mice treated with anti‐PD‐L1 antibody or isotype control antibody. A–F) Data are mean values ± SEM representative of two independent experiments (*n* = 6). *p < 0.05, **p < 0.005, ***p < 0.001, ****p < 0.0001 (two‐way ANOVA).

Anti‐PD‐L1 immunotherapy reinvigorates the antitumor CD8^+^ T cell response for tumor control.^[^ ^23^ ^]^ We wondered whether the improved antitumor CD8^+^ T cell response by NK‐specific *Tipe2* deletion could further improve the therapeutic efficacy of anti‐PD‐L1 immunotherapy. We found that NK‐specific *Tipe2* deletion alone displayed comparable or even slightly better tumor control over anti‐PD‐L1 immunotherapy in control mice (**Figure** **6**A,B). Anti‐PD‐L1 immunotherapy combined with NK‐specific *Tipe2* deletion further reduced the growth of both MC38 and B16 tumors in *Tipe2^ΔNK/ΔNK^* mice (Figure 6A,B). These data suggest that TIPE2 deficiency in NK cells might synergize with anti‐PD‐L1 immunotherapy to further promote the antitumor CD8^+^ T cell response. To further reveal the mechanisms underlying the helper functions of NK cells in CD8^+^ T cell‐dependent anti‐PD‐L1 immunotherapy, we set out to determine tumor growth after anti‐PD‐L1 immunotherapy in mice with NK cell‐specific deletion of *Prf1*, *Ifng*, *Tbx21*, or *Eomes*. We found that mice with NK‐specific *Prf1*, *Ifng*, or *Tbx21* deficiency failed to respond to anti‐PD‐L1 immunotherapy (Figure 6C–E), indicating that T‐bet‐dependent NK cell effector functions are essential for an optimal response to anti‐PD‐L1 immunotherapy. On the other hand, anti‐PD‐L1 immunotherapy induced similar tumor‐suppressive effects in *Eomes^ΔNK/ΔNK^* mice as in control mice (Figure 6F), indicating that NK‐expressed Eomes is required for NK cell support in the antitumor CD8^+^ T cell response but not for further response to anti‐PD‐L1 immunotherapy. Taken together, these data demonstrated that NK‐expressed T‐bet‐dependent effector functions are required for optimal therapeutic efficacy of anti‐PD‐L1 immunotherapy, which could be improved by *Tipe2* deletion in NK cells.

## 3 Discussion

Productive T cell antitumor immunity is initiated and coordinated by antitumor innate immunity.^[^ ^4^, ^5^, ^24^ ^]^ Limited benefits from current T cell‐based checkpoint immunotherapy suggest suboptimal antitumor T cell responses both with and without checkpoint immunotherapy. Such defects are consistent with our poor understanding of T cell‐intrinsic exhaustion mechanisms, as well as with the limited knowledge on the support of other lymphocytes to T cells, especially those from the innate immune system.

Our data demonstrated that NK cells are not only potent antitumor effector lymphocytes themselves but also help to support adaptive antitumor immunity. Previous studies have revealed the essential role of NK cell helper function in infectious immunity. For example, a lack of NK cells increases viral persistence and reduces virus‐specific CD8^+^ T cells in a mouse model mimicking acute HBV infection.^[^ ^25^ ^]^ Adoptive transfer of NK cells reverses the weakened antiviral CD8^+^ T cell response in high‐dose influenza virus infection^[^ ^26^ ^]^ and in the acute HBV infection model.^[^ ^25^ ^]^ NK cells also rescue the CD8^+^ T cell response against infections in the absence of CD4^+^ T cells.^[^ ^27^, ^28^ ^]^ However, very few studies have investigated the molecular mechanisms of NK cell helper function in adaptive antitumor immunity, as well as in antitumor checkpoint immunotherapy. Here, by using the MC38 mouse colon cancer model frequently employed to study the antitumor T cell response, as well as anti‐PD‐1/PD‐L1 immunotherapy, we revealed the helper role of NK cells in the antitumor T cell response by showing that the T‐bet/Eomes‐IFN‐*γ* axis in NK cells is essential for CD8^+^ T cell‐dependent tumor control and that T‐bet‐dependent NK cell effector functions are required for an optimal response to anti‐PD‐L1 immunotherapy. Importantly, we also showed that NK cell‐expressed TIPE2 represents an intracellular checkpoint molecule for NK cells, whose deletion not only enhances NK‐intrinsic antitumor activity but also indirectly supports the antitumor CD8^+^ T cell response and synergizes with anti‐PD‐L1 immunotherapy.

By using an NK‐specific *Tipe2* knockout mice, in which *Tipe2* gene expression was deficient in NK cells with no reduction in the NK cell population size, we previously showed that *Tipe2* ^−/−^ mouse NK cells produce higher levels of IFN‐*γ* than control mouse NK cells in the tumor.^[^ ^19^ ^]^ In this study, we further found that tumor‐infiltrating *Tipe2* ^−/−^ mouse NK cells increased the expression of T‐bet and Eomes. The roles of T‐bet and Eomes in promoting IFN‐*γ* expression^[^ ^2^, ^3^ ^]^ suggest that loss of TIPE2 might facilitate IFN‐*γ* expression through upregulation of T‐bet and Eomes. Furthermore, soluble IL‐15/IL‐15R*α* complexes (sIL‐15 complexes) are abundant in the tumor microenvironment, enabling productive antitumor immunity.^[^ ^29^, ^30^ ^]^ We previously showed that loss of TIPE2 enhances IL‐15‐mTOR signaling in NK cells.^[^ ^19^ ^]^ Considering the role of the IL‐15‐mTOR axis in the induction of the expression and activity of T‐bet and Eomes,^[^ ^31^, ^32^, ^33^, ^34^, ^35^ ^]^ loss of TIPE2 might therefore benefit the IL‐15‐mTOR‐T‐bet/Eomes signaling axis of NK cells in the tumor microenvironment for improved effector functions, as featured by increased IFN‐*γ* production and perforin expression we showed in this study and our previous study.^[^ ^19^ ^]^ Increased cytolytic activity of *Tipe2* ^−/−^ tumor‐infiltrating NK cells might cause the release of more tumor antigen, which could be taken up by DCs to promote the priming of CD8^+^ T cells. In addition, increased IFN‐*γ* production by *Tipe2* ^−/−^ tumor‐infiltrating NK cells might not only facilitate DC maturation by enhanced killing of immature DCs but also upregulate MHC I expression on tumor cells for better recognition by tumor antigen‐specific CD8^+^ T cells. Together these potential mechanisms might contribute to the improved helper function of *Tipe2* ^−/−^ NK cells for the enhanced antitumor CD8^+^ T cell response we observed.

In addition to the accumulating evidence showing the role of immune cells‐expressed TIPE2 as an intracellular checkpoint molecule in antitumor immunity, it should be noted that the role of nonimmune cells‐expressed TIPE2 might play an opposite role to suppress tumor growth and possibly to support antitumor immunity. For example, tumor cell‐expressed TIPE2 has been reported to suppress tumor growth.^[^ ^36^, ^37^, ^38^ ^]^ Furthermore, hydrodynamic gene delivery of TIPE2 results in the activation of antitumor immunity, including the activation of NK cells.^[^ ^36^ ^]^ Since the approach of hydrodynamic gene delivery causes gene expression mainly in liver parenchymal cells (hepatocytes),^[^ ^39^, ^40^ ^]^ the study suggests that hydrodynamic gene delivery of TIPE2 might indirectly activate the antitumor immune response via overexpressing TIPE2 in nonimmune cells.

In summary, we demonstrated that NK cells provide essential help for an optimal antitumor CD8^+^ T cell response and for an optimal response to anti‐PD‐L1 checkpoint immunotherapy. During this process, TIPE2, a previously identified checkpoint molecule in NK cell maturation and tumor immunity, also represents a checkpoint regulating NK cell helper function in supporting the antitumor CD8^+^ T cell response. These results shed light on the importance of NK cells in tumor immunity and suggest that targeting NK cell helper function might be a promising strategy to synergize with T cell‐based immunotherapy for improved tumor control.

## 4 Experimental Section

### Mice

*Tipe2^flox/flox^* mice were purchased from the Cam‐Su Genomic Resource Center (Suzhou, China). *Prf1^flox/flox^* mice, *Ifng^flox/flox^* mice, and *Tipe2* reporter mice with a *Tipe2‐ires‐Egfp* knock‐in cassette were generated by Cyagen (Suzhou, China). *Tbx21^flox/flox^* mice and *Eomes^flox/flox^* mice were purchased from the Jackson Laboratory (Bar Harbor, USA). *Ncr1*‐iCre mice were purchased from Biocytogen (Beijing, China). NK‐specific gene‐deficient mice (such as “*Tipe2* ^ΔNK/ΔNK^ mice”) were generated by crossing flox mice with *Ncr1*‐iCre mice. *Tipe2* ^ΔNK/ΔNK^ mice were further crossed with *Prf1^flox/flox^, Ifng^flox/flox^, Tbx21^flox/flox^*, or *Eomes^flox/flox^* mice to generate NK‐specific double gene‐deficient mice. All mice used were 5 to 8 weeks old with a C57BL/6 background and were housed in the specific pathogen‐free facility at the Shenzhen Institute of Advanced Technology, Chinese Academy of Sciences. All animal experiments were approved by the Institutional Animal Care and Use Committee with an approval number of SIAT‐IACUC‐20221204‐HCS‐MYZX‐BJC‐A2020‐03.

### Cell Lines

MC38 colon cancer cells were gifts from Dr. Yang‐xin Fu (Tsinghua University). B16 melanoma cells and YAC1 lymphoma cells were purchased from Shanghai Cell Bank of the Chinese Academy of Sciences (Shanghai, China). For the generation of MC38‐OVA cells, a pLVX‐CMV‐IRES‐EGFP vector containing an ovalbumin protein‐encoding sequence was generated by GENEWIZ (Burlington, USA). Lentiviral supernatant was generated by co‐transfecting HEK293T cells with the above plasmid along with pMD2.G and psPAX2 plasmids using Lipofectamine 3000 reagent (Invitrogen, Carlsbad, USA).^[^ ^41^ ^]^ Culture supernatants collected 24 h after transfection were added directly to MC38 cells plated in 96‐well culture plates for 12 h before replaced with fresh complete Dulbecco's modified Eagle medium (DMEM). EGFP^+^ cells among transduced cells were sorted as EGFP^+^ MC38‐OVA cells at least 1 week after transduction. pMD2.G (Addgene plasmid # 12259; http://n2t.net/addgene:12259; RRID:Addgene_12259) and psPAX2 (Addgene plasmid # 12260; http://n2t.net/addgene:12260; RRID:Addgene_12260) were gifts from Didier Trono.

### Tumor Models

Groups of six mice per experiment were used. The group size ensured enough power to determine biological differences. No mice were excluded from this study, and no active randomization was applied to groups. The investigators were not blinded to group allocation during the experiment and/or when assessing the outcome. Single‐cell suspensions of MC38 (2 × 10^5^ cells per mouse), MC38‐OVA (5 × 10^5^ cells per mouse), or B16 (7 × 10^4^ cells per mouse) cells were injected subcutaneously into the right flank of the indicated strains of mice.

For depletion of NK cells, mice were intraperitoneally injected with anti‐NK1.1 (PK136) antibody or mouse IgG2*α* isotype control 1 day before tumor challenge. For depletion of CD8^+^ T cells, mice were intraperitoneally injected with anti‐CD8 (53‐6.7) antibody or rat IgG2*α* isotype control on day 1 and day 3 of tumor challenge.

For adoptive transfer of NK cells, WT or *Tipe2‐*deficient mouse NK cells were enriched by a mouse NK Cell Isolation Kit (Miltenyi Biotec, Bergisch Gladbach, Germany). Purity of CD3^−^NK1.1^+^ NK cells was usually above 80% as determined by flow cytometry. 2.5 × 10^5^ WT or *Tipe2‐*deficient mouse NK cells ex vivo stimulated for 2 days with 20 ng mL^−1^ IL‐12 (Peprotech, Cranbury, USA), 50 ng mL^−1^ mouse IL‐15/IL‐15R complex (Thermo Fisher, Waltham, USA), and 10 ng mL^−1^ IL‐18 (Peprotech, Cranbury, USA) were subcutaneously injected into the peritumoral region 3 days after tumor challenge.

For adoptive transfer of OT1 CD8^+^ T cells, 10^5^ CD62^+^CD44^−^ naïve TCR V*α*2^+^CD8^+^ T cells purified from the spleens of OT1 mice were intravenously injected into the mice after tumor challenge on the same day.

### Cell Isolation

Mice were euthanized for analysis of TdLN cells or tumor infiltrating lymphocytes on days 16 to 21 following tumor injection, unless indicated otherwise. Tumor tissue was cross‐cut and minced into small pieces for digestion by 1 mg mL^−1^ collagenase I (Sigma‐Aldrich, St. Louis, USA) dissolved in DMEM in a 37 °C shaker for 45 min. Separated single cells were filtered through cell strainers (BD Biosciences, San Diego, USA). Filtered cells were subjected to Percoll (GE, Boston, USA) density gradient centrifugation to obtain lymphocyte‐enriched compartments for subsequent use in flow cytometry or single‐cell RNA sequencing.

### Antibodies and Flow Cytometry

Single‐cell suspensions of tumor‐infiltrating lymphocytes or TdLN cells were stained with the appropriate monoclonal antibody in phosphate‐buffered saline containing 10% rat serum. When necessary, intracellular staining was performed using the TrueNuclear Transcription Factor Buffer Set (BioLegend, San Diego, USA) according to the manufacturer's instructions. CytoFLEX (Beckman Coulter, Brea, USA) and FACSAria III (BD Biosciences, San Diego, USA) were used for analysis and cell sorting, respectively. Dead cells were excluded by the LIVE/DEAD Fixable Violet Dead Cell Stain Kit (Invitrogen, Carlsbad, USA). Antibodies specific for mouse CD3 (17A2), IFN‐*γ* (XMG1.2), CD8*α* (53‐6.7), TNF‐*α* (MP6‐XT22), NK1.1 (PK136), CD45 (30‐F11), CD69 (H1.2F3), perforin (S16009A), TCR V*α*2 (B20.1), and T‐bet (4B10) were purchased from Biolegend (San Diego, USA). Antibodies specific for mouse Eomes (Dan11mag) were purchased from Thermo Fisher (Waltham, USA). For determination of intracellular IFN‐*γ* expression, isolated single‐cell suspensions of tumor‐infiltrating lymphocytes or TdLN cells were stimulated with Cell Stimulation Cocktail (Thermo Fisher, Waltham, USA) for 3 h in complete RPMI 1640 medium before antibodies staining.

### In Vitro Cytolytic Assay

For cytolytic assays against YAC1 cells, mouse CD3^−^NK1.1^+^ NK cells were sorted from isolated tumor infiltrating lymphocytes. Purity is usually above 95% as determined by flow cytometry. Cell Trace Violet (CTV, Invitrogen, Carlsbad, USA)‐labeled YAC1 target cells were cocultured with NK cells at the indicated effector:target (E:T) ratios for 4 h. After that, cell mixtures were stained with 7‐AAD (Biolegend, San Diego, USA) to determine the percentages of 7‐AAD^+^ dead cells among CTV^+^ target cells.

### Statistical Analysis

Statistically significant differences between two groups were determined by Student's *t*‐tests or one‐way analysis of variance (ANOVA) when appropriate or by two‐way ANOVA in the comparison of tumor growth. *p* < 0.05 was considered significant in all analyses.

## Conflict of Interest

The authors declare no conflict of interest.

## Author Contributions

Conceptualization: J.B., Z.T., H.S.; Methodology: X.J., C.Z., C.H., C.Z., X.Z.; Investigation: J.B., Z.T., H.S.; Writing: J.B., Z.T., H.S.; Supervision: J.B., Z.T., H.S.

## Data Availability

The data that support the findings of this study are available from the corresponding author upon reasonable request.
